# Budget Impact Analysis of Diabetes Drugs: A Systematic Literature Review

**DOI:** 10.3389/fpubh.2021.765999

**Published:** 2021-11-19

**Authors:** Zejun Luo, Zhen Ruan, Dongning Yao, Carolina Oi Lam Ung, Yunfeng Lai, Hao Hu

**Affiliations:** ^1^State Key Laboratory of Quality Research in Chinese Medicine, Institute of Chinese Medical Sciences, University of Macau, Taipa, Macao SAR, China; ^2^Department of Public Health and Medicinal Administration, Faculty of Health Sciences, University of Macau, Taipa, Macao SAR, China; ^3^School of Public Health and Management, Guangzhou University of Chinese Medicine, Guangzhou, China

**Keywords:** budget impact analysis, diabetes, cost-effectiveness, BIA, CEA

## Abstract

**Background:** Budget impact analysis (BIA) is an economic assessment that estimates the financial consequences of adopting a new intervention. BIA is used to make informed reimbursement decisions, as a supplement to cost-effectiveness analyses (CEAs).

**Objectives:** We systematically reviewed BIA studies associated with anti-diabetic drugs and assessed the extent to which international BIA guidelines were followed in these studies.

**Methods:** We conducted a literature search on PubMed, Web of Science, Econlit, Medline, China National Knowledge Infrastructure (CNKI), Wanfang Data knowledge Service platform from database inception to June 30, 2021. ISPOR good practice guidelines were used as a methodological standard for assessing BIAs. We extracted and compared the study characteristics outlined by the ISPOR BIA Task Force to evaluate the guideline compliance of the included BIA.

**Results:** A total of eighteen studies on the BIA for anti-diabetic drugs were identified. More than half studies were from developed countries. Seventeen studies were based on model and one study was based on real-world data. Overall, analysis considered a payer perspective, reported potential budget impacts over 1–5 years. Assumptions were mainly made about target population size, market share uptake of new interventions, and scope of cost. The data used for analysis varied among studies and was rarely justified. Model validation and sensitivity analysis were lacking in the current BIA studies. Rebate analysis was conducted in a few studies to explore the price discount that was required for new interventions to demonstrate cost equivalence to comparators.

**Conclusion:** Existing studies evaluating budget impact for anti-diabetic drugs vary greatly in methodology, some of which showed low compliance to good practice guidelines. In order for the BIA to be useful for assisting in health plan decision-making, it is important for future studies to optimize compliance to national or ISPOR good practice guidelines on BIA. Model validation and sensitivity analysis should also be improved in future BIA studies. Continued improvement of BIA using real-world data is necessary to ensure high-quality analyses and to provide reliable results.

## Introduction

Diabetes is one of the fastest-growing global health emergencies of the twenty-first century and has reached alarming levels, which is associated with significant clinical and economic burdens on society and people with diabetes (1). According to the latest report in the International Diabetes Federation Diabetes Atlas, it was estimated that 463 million people have diabetes in 2019 and this number was projected to reach 578 million by 2030. The total diabetes-related health expenditure was estimated to USD 760 million in 2019 and was projected to increase to USD 825 billion by 2030 (2). The total number of patients with diabetes in mainland China was estimated to be 129.8 million (70.4 million men and 59.4 million women), ranking first in the world, accounting for more than a quarter of the total number of adults with diabetes in the world (3). The high prevalence of diabetes and its risk of complications bring substantial economic burden to patients and their families, and to the health systems and society (4).

Diet and exercise are first line treatments along with metformin to achieve the goal of improving glycemic control and preventing both microvascular and macrovascular complications (5). In order to improve glycemic control in adults with diabetes and reduce the economic burden of diabetes and its complications, new hypoglycemic drugs were constantly developed and applied, including insulin (such as insulin degludec), glucagon-like peptide-1receptor agonist (GLP-1 RA), new oral hypoglycemic agents such as sodium-glucose co-transporter 2 inhibitors (SGLT-2i) and dipeptidyl peptidase-4 inhibitors (DPP-4i).

Budget impact analysis (BIA) addresses the expected changes in the expenditure of a healthcare system after the adoption of a new intervention. It estimates the financial consequences of adoption and diffusion of a new healthcare intervention within a specific healthcare setting given budget constraints. The structure of BIA can be adjusted according to different needs for different countries as well as for time horizons, perspective and underlying diseases (6). Budget impact analyses are an essential part of a comprehensive economic assessment of a health care intervention and are increasingly required by reimbursement authorities as part of a listing or reimbursement submission (7). The ISPOR Task Force developed good practice guidelines to improve high-quality BIAs (7). At the same time, many countries and regions presented specific guidelines (7–9).

As far as we know, there has been no review examined BIA studies in the field of diabetes. Since the high prevalence of diabetes and high treatment costs have a significant impact on drug availability and the sustainability of the reimbursement fund, it is important to study the financial budget for diabetes drugs. Therefore, focusing on the BIA of antidiabetic drugs, this study aimed to review the findings of the current BIA studies and assess the extent to which international BIA guidelines were followed in these studies.

## Methods

### Research Design

This systematic review was conducted according to the Preferred Reporting Items for Systematic Reviews and Meta-Analyses (PRISMA) statement.

### Search Strategy

Based on published guidelines for BIA and other published methodological studies, we conducted a literature search in four English databases (including PubMed, Econlit, Medline, and Web of Science) to identify studies on BIA of antidiabetic drugs published in English or Chinese from 1980 to June 30, 2021. The key concepts used for the search were “budget impact analysis” AND “diabetes mellitus” (see Appendix Tables 1–5). The following search strategy was used: {(Budget impact^*^ OR budgetary impact^*^ OR budget impact analy^*^ OR budgetary impact analy^*^ OR budget impact stud^*^ OR budgetary impact stud^*^) OR [(financial impact^*^ OR economic impact^*^ OR economic analy^*^) AND budget^*^]} AND (diabetes OR diabetes mellitus OR DM OR diabetic). Targeted keyword search was also conducted in two Chinese databases (China National Knowledge Infrastructure (CNKI) and Wanfang Data knowledge Service platform) to identify studies which were published between 1994 and June 30, 2021 and reported estimates of the budget impact of the introduction of a new drug to the treatment.

### Eligibility Criteria

We included studies reporting results of an original BIA pertaining to antidiabetic drugs. Comments, letters, editorials, and meeting abstracts were excluded. We also excluded studies that were not related to diabetes or anti-diabetic drugs, or studies that conducted only cost-effectiveness analyses, reviews, and BIAs of other non-drug interventions for diabetic patients.

### Literature Selection

**Figure 1:**
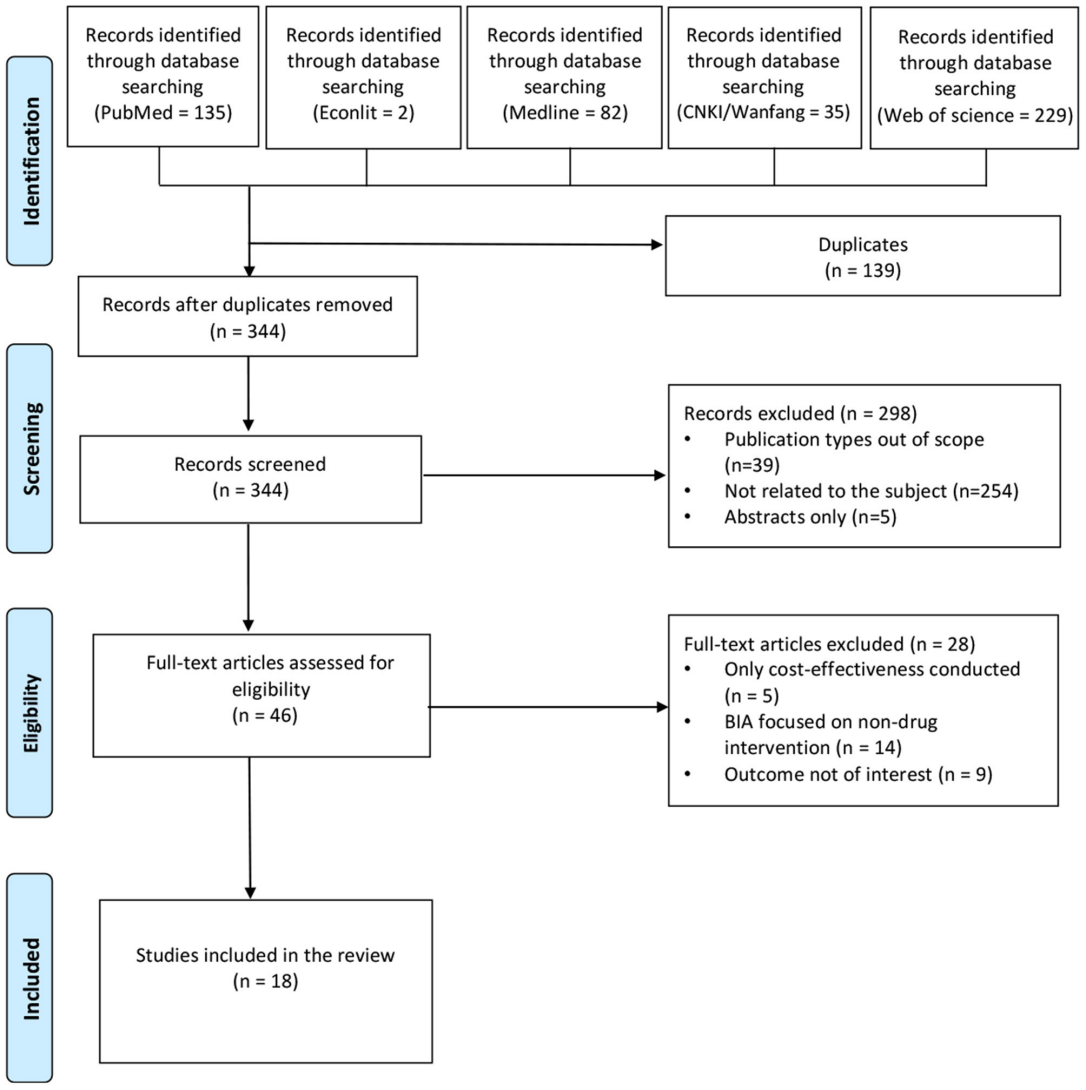
PRISMA flowchart of literature search and selection of publications.

We conducted the duplicate removal and filtering of studies first. Then two reviewers screened the title and abstracts followed by a second round of examining abstracts and full texts independently. A third author resolved any disagreement. This selection process was documented in a PRISMA flowchart (see Figure 1).

### Data Extraction

Based on the ISPOR Task Force guidelines, we developed evidence tables which presented a summary of how each study addressed the key items of the study, including population size and characteristics, budget holder's perspective, budget time horizon, intervention and comparators, market share, model structure, clinical and cost data, cost calculation, and sensitivity analysis. We then systematically extracted data and summarized the findings from all included studies in the evidence tables.

### Guideline Compliance

Assessment of guideline compliance of the included studies was conducted to check the extent to which the ISPOR Task Force guideline of BIA were followed in these studies (7). The assessment was conducted independently by two authors. Any divergence was resolved through discussion and subject to confirmation by another author.

## Results

### Characteristics of Literature Included

Figure 1 summarized the search strategy and the results. Four hundred and eighty three articles were initially retrieved from the search using the keywords about the BIA of antidiabetic drugs. After removal of duplication (*n* = 139), and by screening of title and abstract (*n* = 298) and full text (*n* = 28), 18 BIA studies (10–27) were finally included.

Table 1 summarized the general information of the included BIA studies. More than half of the studies (*n* = 11) were from Europe and the U.S., of which five studies were conducted in US (20, 24–27), two in Italy (14, 22), one in Netherlands (19), one in Spain (18), one in England (13)and one in Bosnia and Herzegovina (15). Apart from these, three studies were conducted in China (10–12), two in Brazil (21, 23), one in Egypt (17) and one in Thailand (16). Among the 18 studies, most of them (*n* = 11) were conducted from a payer's perspective and a few studies (*n* = 5) from the perspective of health care system, while one study (17) was conducted from both of the payer and social perspectives. Besides, the research perspective was not reported in one article (13). All the BIA studies were conducted based on models with one exception (13) that was conducted based on retrospective real-world data. The majority of studies (*n* = 15) focused on BIA only, while the other studies (*n* = 3) combined the BIA with cost-effectiveness analysis.

**Table 1:** General information of the included BIAs.

**References**	**Year**	**Country**	**Sponsored by pharmaceutical industries**	**Perspective**	**Research type**	**Research foundation**	**Intervention**	**Target population**	**Size of target population**
Wehler et al. (27)	2020	United State	Yes^a^	Payer	BIA only	Model	Oral semaglutide	T2DM uncontrolled with metformin and received sitagliptin treatment	1,993 cases in a health plan of 1 million members; and 662,835 cases nationwide
Gout-Zwart et al. (19)	2020	Netherlands	Yes	Payer	BIA only	Model	Metformin SR	T2DM used metformin IR but suffered from AEs and newly diagnosed T2DM	640,000 metformin IR treatment cases and 67,000 newly cases
Laranjeira et al. (21)	2016	Brazil	No	Public health system	BIA only	Model	Long-acting insulin analogs	T1DM	621,941~640,918 cases in 2015~2019
Catic et al. (15)	2017	Bosnia and Herzegovina	NR	Payer	BIA only	Model	Linagliptin	T2DM with DPP-4i treatment	2,624 cases
Marga et al. (18)	2017	Spain	Yes^a^	National Healthcare Service	BIA only	Model	Continuous subcutaneous insulin infusion (CSII)	T1DM that experienced recurrent severe hypoglycemia episodes	NA
Elsisi et al. (17)	2020	Egypt	Yes	Payer & society	BIA only	Model	Dapagliflozin	T2DM	2,053,908 cases for societal perspective and 1,207,698 cases for payer's perspective
Deerochanawong et al. (16)	2018	Thailand	Yes^a^	Payer	BIA only	Model	Biphasic insulin aspart30 (BIAsp 30)	T2DM who needed insulin	0.79 million, 1.77 million, 7.51 million cases in year 1 to year 3
Stefano et al. (14)	2015	Italy	Yes	National Healthcare Service	BIA only	Model	Liraglutide	T2DM patients receiving GLP-1, DPP-4i and SGLT-2i treatments	269,813 cases
Lane et al. (20)	2018	United State	Yes^a^	payer	BIA only	Model	Insulin degludec	T1DM_BBT_ and T2DM_BOT_	1,662 T1DM_BBT_ and 10,602 T2DM_BOT_ in a health plan with 1 million members
Nita et al. (23)	2012	Brazil	Yes^a^	Private health care system	CEA & BIA	Model	Saxagliptine	T2DM with uncontrolled blood glucose on metformin	A health plan with 1 million individuals
Saunders et al. (24)	2014	United State	Yes^a^	Payer	CEA & BIA	Model	Stepwise addition (SWA) of bolus insulin	T2DM patients intensifying to FBB or SWA	6,015 cases in a health plan with 1 million members
Shah et al. (25)	2018	United State	Yes^a^	Payer	CEA & BIA	Model	Liraglutide	T2DM that received GLP-1 treatment	1,130, 1,287, 1,518, 1,762 and 1,937 cases in year 1 to year 5, in a health plan with 1 million members
Weatherall et al. (26)	2017	United State	Yes^a^	Payer	BIA only	Model	Insulin degludec	T1DM_BBT_,T2DM_BOT_ and T2DM_BBT_	59,780 T1DM_BBT_,383,145 T2DM_BOT_ and 171,325 T2DM_BBT_ in a health plan with 35 million members
Xuan et al. (12)	2019	China	NR	Payer	BIA only	Model	Benaglutide	T2DM received treatments	23.4, 23.6, 23.8, 23.9 and 24.0 million cases in 2019~2023
Liu et al. (11)	2018	China	NR	Payer	BIA only	Model	Dapagliflozin	T2DM received treatments	33.06, 33.24, 33.41, 33.58, and 33.75 million patients in 2018–2022
Guan et al. (10)	2016	China	NR	Payer	BIA only	Model	Vildagliptin	T2DM received OAD treatments	4.15, 4.60, 5.09, 5.64, 6.26, and 6.95 million patient-years in 2015–2020
Napoli et al. (22)	2020	Italy	Yes^a^	National Healthcare Service	BIA only	Model	Insulin glargine U300	T2DM insulin-naïve patients	55,318 cases
Agirrezabal et al. (13)	2020	England	NR	NR	BIA only	RWD	Insulin glargine biosimilars (Abasaglar®)	NA	NA

*BIA, Budget impact analysis; CEA, cost effectiveness analysis; T1DM, Type 1 diabetes mellitus; T2DM, Type 2 diabetes mellitus; NA, Not available; NR, Nor report; T1DM_BBT_, Type 1 diabetes mellitus patients on basal-bolus therapy; T2DM_BBT_, Type 2 diabetes mellitus patients on basal-bolus therapy; T2DM_BOT_, Type 2 diabetes mellitus patients on basal-oral therapy; FBB, Full basal-bolus; SWA, Stepwise addition; OAD, Oral antidiabetic drug; RWD, Real world data. AEs, Adverse events; DPP-4i, dipeptidyl peptidase-4 inhibitors; GLP-1: glucagon-like peptide-1; SGLT-2i, sodium-glucose co-transporter 2 inhibitors; Metformin SR, Metformin sustained release; Metformin IR, Metformin immediate release*.

a *Co-authored by at least one employee of sponsoring company*.

About half of the BIA studies (*n* = 8) evaluated the budget impact of insulin, including basal insulin (*n* = 5) (13, 20–22, 26), pre-mix insulin (*n* = 1) (16), bolus insulin (*n* = 1) (24). One study focused on the administration route of insulin (continuous subcutaneous insulin infusion vs. multiple daily insulin injections) instead of the specific type of insulin (18). Among the five basal insulin BIA studies, one of them was about insulin glargine biosimilar (13). The rest of BIA studies targeted GLP-1 RA (*n* = 4, including once-weekly semaglutide, oral semaglutide, and benaglutide) (12, 14, 25, 27), DPP-4i (*n* = 3, including vildagliptin, saxagliptin, and linagliptin) (10, 15, 23), SGLT-2i (*n* = 2, both were dapagliflozin) (11, 17) and metformin (*n* = 1) (19).

The eligible population were mainly chosen according to the indication of intervention and the coverage of the payer's plan. The majority of studies (*n* = 13) (10–12, 14–17, 19, 22–25, 27) restricted the target population on type 2 diabetes patients while some targeted type 1 diabetes patients (*n* = 2) (18, 21), or type 1 and type 2 diabetes patients (*n* = 2) (20, 26), and one study did not specify the target population (13). Six studies estimated the eligible population size based on a hypothetical health plan (20, 23–27), of which the assumed size varied widely ranging from 1 million to 35 million. All but one (23) of these studies reported the specific number of target population. Ten studies calculated the target populations based on the total population in the country (10–12, 14–17, 19, 21, 22). The rest two studies did not report the size of target population (13, 18). Among the 15 studies that reported the specific size of target population, more than half of them (*n* = 13) calculated the size based on local epidemiological data, while the other two studies calculated the size based on real world evidence (10, 22). It is worth noting that one study applied the patient-years instead of patient number to measure the size of target population (10). Two other characteristics were checked: nine studies (16, 18, 20–24, 26, 27) reported conflict of interests (50%), 12 studies (14, 16–20, 22–27) reported pharmaceutical company funding (66.7%), and three studies (10–12) contained no details of conflicts of interests or funding sources (16.7%).

### Methodology and Budget Results of BIAs

Table 2 summarized the methodology and budget results of the included BIA studies. We refined the key study characteristics such as model structure, budget time horizon, discounted rate, treatment strategy, market share of new intervention, cost, and sensitivity analysis methods (13, 17). Among the 17 studies that were based on models, 15 studies used the cost calculation model, and the remaining two studies used Markov model (24), and International T2DM Budget Impact Model (16), respectively.

**Table 2:** Methodology and budget results of BIAs.

**References**	**Model structure**	**Time horizon**	**Discounted rate**	**Treatment strategy (intervention 1 vs. intervention 2)**	**Uptake of new intervention**	**Market share of new intervention**	**Scope of cost**	**Price discount of drug**	**Sensitivity analysis**	**Incremental budget impact value (intervention 1–intervention 2)**
Wehler et al. (27)	Cost calculation based on IQVIA CDM	5 years	No	Oral semaglutide 14 mg vs. Oral sitagliptin 100 mg	Substitution	14, 25, 50, 75, and 100%	Cost of drug, hypoglycemia and complications	base case: 35.1% discount for semaglutide, 72.6% discount for sitagliptin; Sensitivity analyses: 40%, 50% and 60% reduction in the WAC cost for oral semaglutide	one-way	Cost per member per month: +$0.08, +$0.14, +$0.28, +$0.41, +$0.55 in year 1 to year 5 Total annual cost: +$4.6million, +$8.3 million, +$16.5million, +$24.8 million, 33.0 million in year 1 to year 5 A 71.6% WAC discount would be required for oral semaglutide 14 mg to generate 5-year per patient costs equal to sitagliptin 100 mg
Gout-Zwart et al. (19)	Cost calculation based on a decision tree model	3 years	No	Metformin SR vs. metformin IR	Substitution	100%	Direct cost, including acquisition cost and condition-related cost	NR	one-way	Total annual cost: –€232,323, –€645,742, –€180,4271 in year 1 to year 3 (cumulative saving of €1,962,335 during 3 years)
Laranjeira et al. (21)	Cost calculation	5 years	No	Long-acting insulin analogs vs. NPH insulin	Substitution	20, 25, 30, 35, and 40%	Cost of insulin	long-acting insulin analog: 62.5% of the regulated price	one-way and multivariate	Total annual cost: +$28.6 million, +$36.0 million, +$43.5 million, +$51.1 million and +$58.8 million in 2015–2019 (cumulative increased $217.9 million during 5 years)
Catic et al. (15)	Cost calculation	3 years	No	With vs. without linagliptin	Substitution	2, 3, and 5%	Cost of drug	No	No	Total annual cost: –€18,194, –€235,570 and –€699,472 in 2016–2018
Marga et al. (18)	Cost calculation	4 years	No	CSII vs. MII	Substitution	100%	Treatment cost (insulin + insulin pumpkin) and cost of hypoglycemic	Sensitivity analysis: −10% monthly cost of the pump kit	one-way	Cost per patient per year: –€ 9,821 Total annual cost of a hypothetical cohort of 100 patients: –€ 982,023
Elsisi et al. (17)	Cost calculation	3 years	No	Dapagliflozin vs. standard of care	Substitution	5, 10, and 15%	Cost of drug and complications	Sensitivity analysis: ± 25% of drug cost	one-way	Total annual cost from societal perspective: -EGP 121 million, -EGP 243 million and -EGP 365 million in year 1 to year 3 (cumulative saving of EGP 731 million during 3 years) total annual cost from payer's perspective: -EGP 71 million, -EGP 143 million, and -EGP 215 million in year 1 to year 3 (cumulative saving of EGP 430 million during 3 years)
Deerochanawong et al. (16)	International T2DM budget impact model	3 years	3.00%	BIAsp 30 vs. BHI 30	Substitution	1.24, 2.48, and 3.72%	Cost of insulin, hypoglycemia and complications	NR	one-way	Cost per patient per year: +$0.97, +$1.96, +$2.9 in year 1 to year 3; Total annual cost: +$771,349, +$151,8218, +$221,6747 in year 1 to year 3
Stefano et al. (14)	Cost calculation	3 years	NR	Increase vs. current use of liraglutide	Substitution	increase: 16, 17, 18% current: 14.52, 13.62, and 13.20%	Treatment cost (drug and needle)	Ex-factory prices were used including discounted prices	No	Cost per patient per year: +€8.04, +€18.18, +€25 in 2014–2016; Total annual cost: +€2.1 million, +€4.9 million, +€6.7 million in 2014~2016
Lane et al. (20)	Cost calculation	1 year	No	Insulin degludec vs. Insulin glargine U100	Substitution	100%	Cost of insulin and hypoglycemia	Rebate scenario analysis	No	Cost per patient per year: +$312 for T1DM_BBT_, +$907 for T2DM_BOT_; Cost per member per month: +$0.04 for T1DM_BBT_, +$0.80 for T2DM_BOT_; Total annual cost: +$0.51 million for T1DM_BBT_, +$9.62 million for T2DM_BOT_; Rebates of 7.3% (T1DM) and 10.6% (T2DM) were required for IDeg to break-even with IGlar at the full list price.
Nita et al. (23)	Cost calculation	3 years	5.00%	With vs. without saxagliptine	Substitution	0.35% in year 1 and 1.95% in year 3	Cost of drug	Sensitivity analysis: ± 25% of drug cost	one-way	Total cumulative cost in 3 years: –R$ 417,958
Saunders et al. (24)	Markov model	1 years (include32 weeks)	3.50%	SWA of bolus insulin vs. full basal-bolus	Substitution	100%	Cost of insulin, hypoglycemia	NR	one-way	Cost per patient per year: –$1,304 at week 32, –$1,612 in year 1 Cost per member per month: –$1.06 Tt week 32, –$0.81, –$0.53, –$0.39, –$0.30, –$0.24 in year 1 to year 5 Total annual cost: –$7.8 million at week 32, –$9.7 million in year 1
Shah et al. (25)	Cost calculation based on a cohort state-transition model	5 years	3%	After LEADER vs. before LEADER	Substitution	After: 47% for each year Before: 47, 46, 41,31, and 25%	Cost of drug, hypoglycemia and complications	NR	No	Cumulative cost in 5 years: cost per patient per year: –$284 cost per member per month: –$0.02 Total cost: –$266,334
Weatherall et al. (26)	Cost calculation	1 year	NR	Insulin degludec vs. Insulin glargine	Substitution	100%	Cost of insulin and hypoglycemia	The IDeg price was based on a 10% premium to IDet	one-way	Cost per patient per year: –$143.7 (–$357.13 for T1DM_BBT_, –$1206.61 for T2DM_BOT_, +$1420.04 for T2DM_BBT_); Total annual cost: -$240 million (–$21.4 for T1DM_BBT_, –$462.3 for T2DM_BOT_, +$243.3 for T2DM_BBT_);
Xuan et al. (12)	Cost calculation	5 years	NR	With vs. without benaglutide	Substitution	1, 1.5, 1.8, 2.2 and 2.6%	Cost of drug, hypoglycemia and adverse disease	NR	No	Total annual cost: –¥169 million, –¥221 million, –¥293 million, –¥372 million, –¥471 million
Liu et al. (11)	cost calculation	5 years	No	With vs. without dapagliflozin	Substitution	0.2, 0.6, 1.1, 2.3, 3.1, and 3.5%	Cost of drug, hypoglycemia and complications	Sensitivity analysis: −10, −15, and −20% of dapagliflozin price	one-way	Total annual cost: +¥71 million, +¥141 million, +¥254 million, +¥187 million and -¥8 million in 2018–2022
Guan et al. (10)	Cost calculation	5 years	NR	With vs. without vildagliptin	Substitution	0.64, 1.02, 1.41, 1.80, and 2.18%	Cost of drug	Sensitivity analysis: −10, −20% of vildagliptin price	One-way	Cost per patient per year: –¥564 total annual cost: –¥4.59million, –¥8.17million, –¥12.50million, –¥17.69million and –¥23.92 million in 2016–2020
Napoli et al. (22)	Cost calculation	1 year (include 24 weeks)	NR	Insulin glargine U300 vs. Insulin degludec	Substitution	Scenarios A: 0% Scenarios B: 61% (current treatment rate) Scenarios C: 80% Scenarios D: 100%	Cost of insulin	NR	No	Total annual cost: scenario B-A: –€1.07 million at 24 weeks, –€2.89 million in year 1; scenario C-B: –€0.33 million at 24 weeks, –€0.90 million in year 1; scenario D-C: –€0.35 million at 24 weeks, –€0.95 million in year 1; scenario D-A: –€1.76 million at 24 weeks, –€4.73 million in year 1, –€5.53 million in year 2
Agirrezabal et al. (13)	/	4 years	NR	Abasaglar® vs. Lantus®	Substitution	NA	Cost of insulin	NR	No	Total savings with Abasaglar®: 1,549, 90,022, 376,834, 437,524 in 2015–2018 (cumulative saving of 900,000 during 4 years) Total missed savings with Abasaglar®: 2.3 million, 9.3 million, 8.9 million, 5.1 million in 2015–2018 (cumulative missed savings of 25.6 million during 4 years)

*CDM, Core diabetes model; WAC, Wholesale acquisition costs; Metformin SR; Metformin sustained release; Metformin IR, Metformin immediate release; NR, Not report; NA, Not available; T1DM_BBT_, Type 1 diabetes mellitus patients on basal-bolus therapy; T2DM_BBT_, Type 2 diabetes mellitus patients on basal-bolus therapy; T2DM_BOT_, Type 2 diabetes mellitus patients on basal-oral therapy; NPH, Neutral Protamine Hagedorn; MII, Multiple daily insulin injections; CSII, Continuous subcutaneous insulin infusion; EGP, Egyptian Pound; BIAsp, Biphasic Insulin Aspart; BHI, Biphasic Human Insulin; SWA, Stepwise addition*.

The budget time horizon was concentrated in 1–5 years, which was mainly in accord with the guidelines and the requirements of the budget holders. The most commonly used of time horizon was 3 years (*n* = 6) (14–17, 19, 23) and 5 years (*n* = 6) (10–12, 21, 25, 27), followed by 1 year (*n* = 4) (20, 22, 24, 26) and 4 years (*n* = 2) (13, 18). Notably, two studies also conducted within time-horizons of 24 weeks and 32 weeks based on the study duration of the clinical trials (22, 24). The discounting rate was not always clearly reported in the included BIA studies. Four studies reported the discounting rate ranging from 3 to 5% (16, 23–25), eight studies (11, 15, 17–21, 27) did not consider the discounting rate in compliance with the ISPOR Task Force guidelines (7). Meanwhile, six studies (10, 12–14, 22, 26) did not mentioned the discounting rate in the articles.

The treatment strategies were clearly described in all the included studies. Most studies (*n* = 10) compared research drug used in two treatment strategies under different scenarios (13, 16, 18–22, 24, 26, 27). Among these studies, one compared the generic drug insulin glargine biosimilar Abasaglar® with the reference listed drug Lantus® (13), one compared different doses of the same drug (18), and one not only compared different drugs but also the dosage regimen (24). Besides, seven studies examined the impact of adding a new drug to the current treatment regimen (10–12, 15, 17, 23, 25), and the remaining study compared the current use trend and the increased use trend of the same drug (14).

In all included studies, the new intervention was assumed to impact the market by substitution of the current treatments. In other words, it was assumed that the new intervention would replace one or more of the current interventions recommended in the clinical practice of diabetes treatment. Most studies (*n* = 17) reported the hypothetical market shares of the new interventions within the time horizon of the study. Among the 17 studies, 11 assumed that the market share of the new intervention increased gradually (10–12, 14–17, 21–23, 27), five assumed that the new intervention replaced 100% of the market share of the current intervention (18–20, 24, 26), and one study (22) assumed different market shares of the new intervention being higher or lower than its current market share.

The scope of costs calculated in the included studies was summarized as treatment-related costs and condition-related costs according to the ISPOR Task Force guidelines. Treatment-related costs mainly included drug acquisition costs and the associated costs such as administration, diagnostic testing and monitoring. Condition-related costs included adverse event costs and complication costs. Seven studies only calculated treatment-related costs only (10, 13–15, 21–23). Eleven studies took into account both treatment-related costs and condition-related costs (11, 12, 16–20, 24–27), of which nine studies considered the cost of hypoglycemia which was further classified into minor and severe hypoglycemia (11, 12, 16, 18, 20, 24–27), six studies considered the cost of diabetes-related complications (11, 16, 17, 19, 25, 27) (mainly including myocardial infarction (MI), stroke, heart failure, heart disease) and only one took the cost of adverse event (including dizziness, vomiting, fatigue and loss of appetite) into account (12).

Of the 18 included studies, 11 studies were subjected to sensitivity analysis (10, 11, 16–19, 21, 23, 24, 26, 27). Ten of them conducted one-way sensitivity analysis (10, 11, 16–19, 23, 24, 26, 27), while one study conducted one-way and multivariate sensitivity analysis (21). Among the studies that performed the one-way sensitivity analysis, parameters such as the cost of severe hypoglycemia, drug price, treatment adherence, prevalence and market share were commonly included.

Regarding the budget impact results, the budget amounts such as the total annual cost (*n* = 16) (10–22, 24, 26, 27) and the cumulative cost (*n* = 6) (13, 17, 19, 21, 23, 25) were presented in all the included studies. Some studies even presented the cost per patient per year (*n* = 8) (10, 14, 16, 18, 20, 24–26) and the cost per member per month in a hypothetical health plan (*n* = 4) (20, 24, 25, 27). Twelve studies (10, 12, 13, 15, 17–19, 22–26) reported that increasing use of new drug or introducing a new drug into the reimbursement list would reduce the financial budget. Six studies (11, 14, 16, 20, 21, 27) concluded that the adoption or increasing use of new intervention for diabetes treatment would increase the budget. Deerochanawong et al. (16) reported that the adoption of Insulin Aspart 30 instead of Biphasic Human Insulin 30 for people with T2DM in Thailand resulted in additional acquisition cost which was partially offset by reducing the cost of hypoglycemia. Two studies (20, 27) conducted the rebate scenario analysis to estimate the rebate rate or discount rate that was required for a new intervention to generate equal budget impact of the old intervention. Wehler et al. (27) reported that a 71.6% cost discount would be required for oral semaglutide 14 mg to generate 5-year per patient costs equal to sitagliptin 100 mg in US. Lane et al. (20) also reported that rebates of 7.3 and 10.6% at the full list price were required for insulin degludec to break-even with insulin glargine for patients with T1DM and T2DM, respectively.

### Guideline Compliance of the Included Studies

Table 3 provides a summary of the compliance of the included studies to the ISPOR Task Force guidelines (7). The compliance of methodology used the included BIA studies with the ISPOR Task Force guidelines indicates that the included studies were deemed appropriate in terms of perspective, hypothetical scenario, comparator and data sources. Nine studies complied with at least 8 of the 9 items in the guidelines (≥88.9%) (10, 11, 16, 17, 19, 20, 24, 26, 27), and six studies (12, 15, 21–23, 25) complied with seven items (77.8%). Only one study (13) complied with fewer than five items (44.4%). Overall, most studies did not report model validation, and only 22.2% of the studies conducted model validation.

**Table 3:** Guideline compliance of the included studies.

**References**	**Perspective**	**Target population estimate**	**Time horizon**	**Hypothetical scenario**	**Comparator**	**Framework description**	**Data collection and sources**	**Validation**	**Sensitivity analysis**
Wehler et al. (27)	√	√	√	√	√	√	√	√	√
Gout-Zwart et al. (19)	√	√	√	√	√	√	√	√	√
Laranjeira et al. (21)	√	√	√	√	√		√		√
Catic et al. (15)	√	√	√	√	√	√	√		
Marga et al. (18)	√		√	√	√		√		√
Elsisi et al. (17)	√		√	√	√	√	√	√	√
Deerochanawong et al. (16)	√	√	√	√	√	√	√		√
Stefano et al. (14)	√	√	√	√	√		√		
Lane et al. (20)	√	√	√	√	√	√	√	√	
Nita et al. (23)	√	√	√	√		√	√		√
Saunders et al. (24)	√	√	√	√	√	√	√		√
Shah et al. (25)	√	√	√	√	√	√	√		
Weatherall et al. (26)	√	√	√	√	√	√	√		√
Xuan et al. (12)	√	√	√	√	√	√	√		
Liu et al. (11)	√	√	√	√	√	√	√		√
Guan et al. (10)	√	√	√	√	√	√	√		√
Napoli et al. (22)	√	√	√	√	√	√	√		
Agirrezabal et al. (13)			√		√		√		

## Discussion

In this study, we systematically reviewed 18 BIA studies for anti-diabetic drugs for diabetes mellitus, which were conducted in various countries and regions including Europe, the U.S., Asia and South America. The methodological characteristics according to the ISPOR guidelines for BIA (7) and research results were retrieved, summarized and assessed. The primary finding from this review is that despite published guidelines for budget-impact analysis, there were still significant differences in the included studies. In addition to the ISPOR guidelines, many countries and regions had issued budget impact analysis guidelines, such as France (9), Canada (8), Australia (28), and Ireland (29). Although the key elements of budget-impact model design were consistent in these guidelines, the BIA method has not been specified in a unified and standardized form. In our review, most of the health care systems for which the BIA was carried out didn't have their own guidelines except Brazil (30) and the UK (31), but all of these included BIAs were conducted following the ISPOR guidelines.

Major deviations in the study design from the recommendations in the ISPOR Task Force guidelines (7) were the static treated population size, the selected time horizons, the mix of comparators, the limited or the lack of reporting about the validation, and the limited or the lack of sensitivity analysis. These deviations appear to be independent of the interventions in question. The finding of variability in the inclusion of key design elements was also made in the previous reviews of BIAs. Vooren et al. (32) considered that BIA was not a well-established technology in the literature in 2013, and many published studies have not yet reached acceptable quality. Mauskopf (33) found that recommended practice was not followed in many BIAs. Another previous review conducted by Faleiros et al. (34) also considered that most BIA currently conducted were still far from an agreed standard of excellence. Although we agree on the importance of a mature framework for BIA, it was more important to implement the operations of BIA. Such as liraglutide, one study in Italy (14) showed that liraglutide's budget increased, while the BIA study in the U.S. (25) recognized that budget of liraglutide decreased. Similarly, one study in Egypt (17) showed that budget of dapagliflozin decreased, while the BIA study in China (11) recognized that budget of dapagliflozin increased. These may be related not only to the uniform BIA guidelines, but also to the drug reimbursement policy in different countries.

An important recommendation from the ISPOR Task Force guideline (7) is to adopt a model structure as simple as possible. In general, the most commonly used model structure is the cost-calculation model which can indirectly account for these changes in treatment over time through the evolution of treatment shares over time and the related clinical impacts (33). Of the 18 studies included in this review, 14 used a cost-calculation model. Whatever model is used, it should reflect the changes in the resources used and the costs associated with the new intervention as much as possible. We found that some included studies combined the use of the cost-calculation model with other models, such as the IQVIA CORE diabetes model in order to assess the differences in case of chronic diabetic complications and the related costs between the new drug and comparator drugs. Some elements such as time horizon or discounting rates can easily be determined based on the published guidelines or the requirements by decision-makers. ISPOR guidelines suggested that a time horizon of 1–5 years is generally of interest to budget-holders to inform budget planning (7). A time horizon of 3 years is required for National Reimbursement Drug List (NRDL) negotiation submission in China. Mauskofp (33) recommended projections beyond 1 year even if the budget holder was only interested in a 1-year time horizon because the cost and population parameters might change over time. S. Simoens et al. (35) considered that BIA might incorporate future market interactions, competitions, and pricing effects and the stakeholders were increasingly considering long time horizons when contemplating the budget impact of chronic disease therapies. The majority of the studies included in this review determined the time horizon of 3 years or more, while only four studies (20, 22, 24, 26) used a time horizon of 1 year. The discounting rates were not necessarily considered because the time frame of the research was relatively short and the focus was mainly on the real cost in the budgetary year. This may be the reason that the discounting rates were not always clearly reported for the included BIA studies. There were only four studies (16, 23–25) reported the discounting rate which ranged from 3 to 5%.

Some critical study characteristics were difficult to determine, including the estimation of the population size of the new intervention, the determination of comparators, the market share and the selection of model structure. These items are critical because they determine the size of target population for the new intervention, which are the important factors influencing the results of the BIA. Target population are usually estimated by two ways: based on epidemiological data or based on real world evidence. In our review, most studies (72.22%) were conducted based on local epidemiological data. In some countries, such as China, epidemiological data related to diseases and drug treatments are also required for the NRDL submission. In addition, the guidelines of BIA indicated that the target population is not a static group, but a dynamic one that varies with incidence, cure, prognosis, and death. But most studies in this review did not appropriately account for a dynamically changing population while only six studies (12, 14, 15, 19, 21, 23) used the dynamic population assumption. In a previous review, Mauskopf (33) believed that if the increase in the size of the treated population were not taken into account, the resulting budgetary impact estimates were likely to be biased (33).

Another difficulty in conducting BIA is making the assumption of intervention/comparator market uptake. When a new drug is introduced, there are many factors influencing the change in the market share of the new drug and the comparators. It is difficult for budget holders to evaluate the accuracy of these assumptions based on the available evidence and data. So far, many published BIA studies did not take market uptake into account, but assumed the extreme cases instead where the comparator drug was 100% replaced by a new drug. There are three types of market change according to the ISPOR guidelines, including substitution, combination, expansion (36). Our review shows that the substitution was assumed in all the included studies. This is determined by the characteristics of the disease and its treatment.

Our review corroborates precedent findings (32, 33) on limited model validation and sensitivity analysis of current BIAs. Model validation and sensitivity analysis should be carried out to ensure the robustness of the BIA research. A sensitivity analysis is essential to investigate the influence of assumptions on structural aspects or variable inputs of the BIAs (37). Moreover, sensitivity analysis allows a more comprehensive prediction of budget impact. But sensitivity analysis was performed in only 11 of the 18 studies included in our review. In addition, we found that most of the included studies did not perform the model validation. Only two studies (17, 19) stated that the validity of the BIA model was discussed with clinical experts and relevant researchers and two study adopted a verified model (20, 27). Many guidelines had already put forward requirements for the validity verification of the BIA model. Obviously, the compliance of the BIA with the ISPOR guidelines should be improved especially in such areas as sensitivity analysis and model validation.

The features of health care systems should be taken into account when conducting BIA. For example, in China, the reimbursement rate for outpatient and inpatient is different. The reimbursement rate of inpatients (65%) is significantly higher than that of outpatients (50%), which should be taken into account when submitting the BIA for NRDL negotiation. Furthermore, the characteristics of the medical system are also related to the selection of cost range. We found that the studies included in this review varied in terms of treatment-related costs. Some studies not only included the drug acquisition costs, but also analyzed the associated costs, such as needle costs, self-measured blood glucose (SMBG) costs. This might not be applicable in all healthcare settings. For instance, in China, the needle and SMBG related expenses are not reimbursed in most provinces and cities. Therefore, such costs should not be included when a BIA is conducting from the perspective of payer.

The conflict of interest and the funding sources cannot be ignored when considering the quality of BIAs. It should be noted that the vast majority were sponsored by the pharmaceutical companies, and as expected, the authors' conclusions of all sponsored studies were in favor of these drugs. In this way, BIAs have deviated from the intended goal of providing short-term economic consequences from a health system perspective and appeared to be tailored to show short-term savings. In 2016, Faleiros et al. (34) reported the weakness of many current BIA studies might be directly linked to the funding of pharmaceutical companies and conflict of interest. The Vooren et al. (32) review also expressed a concern that most of the published BIAs for European Union countries were sponsored by the drug manufacturer and that this might be a bias of the estimates. In our review of 18 BIAs, 12 were sponsored by industry or had industry authors.

Another contributor to improper quality might be that there have been counterarguments on the usefulness of BIAs due to the close proximity of the technique to CEA (38). However, BIA is not a substitute for cost-effectiveness analysis. They are indeed complementary to each other to support decision making. BIA addresses the financial stream of consequences related to the uptake and diffusion of technologies to assess their affordability. CEA evaluates the costs and outcomes of alternative technologies over a specified time horizon to estimate their economic efficiency. Both CEA and BIA as should be considered as important yet separate components of a comprehensive pharmacoeconomic evaluation of an intervention (36). In our review, the majority of included studies focused on BIA only, while only three studies (23–25) combined the BIA with cost-effectiveness analysis together. For the three studies that presented the results of both cost-effectiveness analysis and budget-impact analysis, we found that the information provided for budget impact model design, assumptions, input and results was insufficient to completely characterize the model. Some detailed information was provided for the cost-effectiveness analysis, some of which was relevant for the budget-impact analysis, while no detailed information was provided on estimated population size, characteristics, and change in the treatment mix. Mauskopf (33) considered that it was critical for the structure, assumptions, and input values for both models to be described in detail in the published study.

### Recommendations for Future BIA for Anti-diabetic Drugs

Unlike rare disease, diabetes is a chronic progressive disease with a high prevalence and a large patient population, especially in patients with type 2 diabetes. Moreover, the incidence and mortality of type 2 diabetes are equivalent, which should be negligible. Therefore, it is acceptable to consider the total population as a static group instead of taking short-term population changes into account when calculating the target population in BIA study.

Chronic complications are the main cause of the heavy economic burden of diabetes. Research showed that 81% of the total medical expenses for T2DM were used for the treatment of diabetes-related complications (39). In addition, hypoglycemia is a common acute complication in the treatment of diabetes mellitus which also brings heavy economic burden. Thus, in the analysis of the budget impact of anti-diabetic drugs, costs should not only be restricted to drug-costs and the cost of hypoglycemic event, the cost of diabetic chronic complications such as cardiovascular disease should also be considered.

The key analytical process and input parameters should be validated when conducting a BIA according to the ISPOR guidelines and the economic evaluation guidelines. Validation could be done by consulting budget holders and corroborating model parameters. All inputs and formulas should be validated by a second budget impact expert. After the new intervention is introduced, it is recommended to continue data collection and compare it with the estimates obtained from the BIA. This will provide important reference for future decision-making and studies. Furthermore, it is suggested to conduct analysis from multiple perspectives including payer, healthcare system for the complete consideration of related costs.

### Strengths and Limitations

This review is the first to assess the methodology and the guideline compliance of BIAs specifically for anti-diabetic drugs. We have summarized the key elements to ensure the quality of BIA research comprehensively. In addition, we concluded the budget results of the included studies to provide a comprehensive reference for BIA studies of antidiabetic drugs. A potential limitation of this review is that we only included studies published in English and Chinese due to the language capacity limitation of the research team. References were retrieved from four international databases and two Chinese databases. (Pub-med, Econlit, Medline, Web of science, CKNI, and Wan-fang Data Knowledge Service Platform of China). Moreover, it should be noted that BIAs directly submitted to reimbursement agencies were not studied.

## Conclusion

BIA is an important tool to assess the affordability of adopting a new antidiabetic drug in a certain health setting amid the rise of many new diabetes drugs. Our systematic review finds that there seems to be great variability in the study design and some studies had low compliance to the ISPOR guidelines. In order to provide useful and high-quality evidence to assist the decision-making process, researcher should ensure their BIA studies were conducted in compliance with the recommended guidelines or the requirements according to the decision makers. Besides, continued improvement of the validity of the model and sensitivity analysis are necessary. Furthermore, the accuracy of parameters in the BIA needs to be more rigorously demonstrated to indicate the quality of the findings. Finally, more BIA studies for antidiabetic drugs based on real-world data should be conducted in future research.

## Data Availability Statement

The original contributions presented in the study are included in the article/Supplementary Material, further inquiries can be directed to the corresponding author.

## Author Contributions

ZL, ZR, and HH conceptualized this study. ZL, ZR, and DY collected the materials. ZL, ZR, and HH conducted analysis. ZL, ZR, CU, YL, and HH drafted the manuscript. All authors reviewed and approved the final vision. All authors contributed to the article and approved the submitted version.

## Funding

This research is partially supported by the grants from the University of Macau (MYRG2020-00230-ICMS).

## Conflict of Interest

The authors declare that the research was conducted in the absence of any commercial or financial relationships that could be construed as a potential conflict of interest.

## Publisher's Note

All claims expressed in this article are solely those of the authors and do not necessarily represent those of their affiliated organizations, or those of the publisher, the editors and the reviewers. Any product that may be evaluated in this article, or claim that may be made by its manufacturer, is not guaranteed or endorsed by the publisher.
