# Supplementary material for: Budget Impact Analysis of Diabetes Drugs: A Systematic Literature Review
Source: Front Public Health. 2021 Nov 19;9:765999. doi: 10.3389/fpubh.2021.765999 (PMC8639520; doi:10.3389/fpubh.2021.765999)
Supplement: Supplementary file 1 [file Table_1.docx]

**Appendix**

Table 1 PubMed

| Search  No. | Search terms | Hits |
| --- | --- | --- |
| Budget-impact studies | | |
| #1 | budget impact*[Title/Abstract] OR budgetary impact*[Title/Abstract] OR budget impact analy*[Title/Abstract] OR budget impact analy*[Text Word] OR budgetary impact analy*[Title/Abstract] OR budgetary impact analy*[Text Word] OR budget impact stud*[Title/Abstract] OR budget impact stud*[Text Word] OR budgetary impact stud*[Title/Abstract] OR budgetary impact stud*[Text Word] OR ((financial impact*[Title/Abstract] OR economic impact*[Title/Abstract] OR economic analy*[Title/Abstract]) AND budget*[Text Word]) Filters: English | 2182 |
| Diabetes | | |
| #2 | ((((diabetes [Title/Abstract]) OR (diabetes [Text Word])) OR (diabetes mellitus [Title/Abstract])) OR (DM[Title/Abstract])) OR (diabetic [Title/Abstract]) Filters: English | 682822 |
| Limits (no comments, letter, editorials, conference abstracts) | | |
| #3 | (("Comment"[Publication Type]) OR ("Letter"[Publication Type])) OR ("Editorial"[Publication Type])) Filters: English | 1846557 |
| Total | | |
| #4 | (#1 AND #2) NOT #3 Filters: English | 135 |

**Appendix**

Table 2 Econlit

| Search  No. | Search terms | Hits |
| --- | --- | --- |
| Budget-impact studies | | |
| #1 | (TI ( "Budget impact*" OR "budgetary impact*" ) OR AB ( "Budget impact*" OR "budgetary impact*" ) OR TI ( "budget impact analy*" OR "budgetary impact analy*" OR "budget impact stud*" OR "budgetary impact stud*" ) OR AB ( "budget impact analy*" OR "budgetary impact analy*" OR "budget impact stud*" OR "budgetary impact stud*" ) OR TX ( "budget impact analy*" OR "budgetary impact analy*" OR "budget impact stud*" OR "budgetary impact stud*" )) OR ((AB ( "financial impact*" OR "economic impact*" OR "economic analy*" ) OR TI ( "financial impact*" OR "economic impact*" OR "economic analy*" )) AND TX budget*) Filters: English | 180 |
| Diabetes | | |
| #2 | TI diabetes OR AB diabetes OR TX diabetes OR AB "diabetes mellitus" OR TI "diabetes mellitus" OR AB diabetic OR TI diabetic OR AB DM OR TI DM Filters: English | 513 |
| Limits (English; no comments, letters, editorials, conference abstracts) | | |
| #3 | SU Comment* OR SU Letter OR SU Editorial OR SU "Meeting Abstracts" Filters: English | 5 |
| Total |  |  |
| #4 | (#1 AND #2) NOT #3 Filters: English | 2 |

**Appendix**

Table 3 Medline

| Search  No. | Search terms | Hits |
| --- | --- | --- |
| Budget-impact studies | | |
| #1 | (TI ( "Budget impact*" OR "budgetary impact*" ) OR AB ( "Budget impact*" OR "budgetary impact*" ) OR TI ( "budget impact analy*" OR "budgetary impact analy*" OR "budget impact stud*" OR "budgetary impact stud*" ) OR AB ( "budget impact analy*" OR "budgetary impact analy*" OR "budget impact stud*" OR "budgetary impact stud*" ) OR TX ( "budget impact analy*" OR "budgetary impact analy*" OR "budget impact stud*" OR "budgetary impact stud*" )) OR ((AB ( "financial impact*" OR "economic impact*" OR "economic analy*" ) OR TI ( "financial impact*" OR "economic impact*" OR "economic analy*" )) AND TX budget*) Filters: English | 980 |
| Diabetes | | |
| #2 | TI diabetes OR AB diabetes OR TX diabetes OR AB "diabetes mellitus" OR TI "diabetes mellitus" OR AB diabetic OR TI diabetic OR AB DM OR TI DM Filters: English | 415268 |
| Limits (English; no comments, letters, editorials, conference abstracts) | | |
| #3 | SU Comment* OR SU Letter OR SU Editorial OR SU "Meeting Abstracts" Filters: English | 4671 |
| Total |  |  |
| #4 | (#1 AND #2 ) NOT #3 Filters: English | 82 |

**Appendix**

Table 4 Web of Science

| Search  No. | Search terms | Hits |
| --- | --- | --- |
| Budget-impact studies | | |
| #1 | (((((TI=(Budget impact* OR budgetary impact*)) OR AB=(Budget impact* OR budgetary impact*)) OR TI=(budget impact analy* OR budgetary impact analy* OR budget impact stud* OR budgetary impact stud*)) OR AB=(budget impact analy* OR budgetary impact analy* OR budget impact stud* OR budgetary impact stud*)) OR TS=(budget impact analy* OR budgetary impact analy* OR budget impact stud* OR budgetary impact stud*) OR (((TI=(financial impact* OR economic impact* OR economic analy*)) OR AB=(financial impact* OR economic impact* OR economic analy*)) AND TS=(budget*)) Filters: English | 29381 |
| Diabetes | | |
| #2 | ((TI=(diabetes OR diabetes mellitus OR diabetic OR DM)) OR AB=(diabetes OR diabetes mellitus OR diabetic OR DM)) OR TS=(diabetes) Filters: English | 819575 |
| Limits (no letter, editorial material, meeting abstracts) | | |
| #3 | ((DT= (Editorial Material)) OR DT=(Letter)) OR DT= (Meeting Abstract) Filters: English | 11717058 |
| Total |  |  |
| #4 | (#1 AND #2) NOT #3 Filters: English | 229 |

**Appendix**

Table 5 Search strategy

| Source | Search syntax | Hits，30 June 2021 |
| --- | --- | --- |
| PubMed | ((Budget impact* OR budgetary impact* OR budget impact analy* OR budgetary impact analy* OR budget impact stud* OR budgetary impact stud*) OR ((financial impact* OR economic impact* OR economic analy*) AND budget*)) AND (diabetes OR diabetes mellitus OR DM OR diabetic) | 135 |
| EconLit | ((Budget impact* OR budgetary impact* OR budget impact analy* OR budgetary impact analy* OR budget impact stud* OR budgetary impact stud*) OR ((financial impact* OR economic impact* OR economic analy*) AND budget*)) AND (diabetes OR diabetes mellitus OR DM OR diabetic) | 2 |
| Medline | ((Budget impact* OR budgetary impact* OR budget impact analy* OR budgetary impact analy* OR budget impact stud* OR budgetary impact stud*) OR ((financial impact* OR economic impact* OR economic analy*) AND budget*)) AND (diabetes OR diabetes mellitus OR DM OR diabetic) | 82 |
| Web of Science | ((Budget impact* OR budgetary impact* OR budget impact analy* OR budgetary impact analy* OR budget impact stud* OR budgetary impact stud*) OR ((financial impact* OR economic impact* OR economic analy*) AND budget*)) AND (diabetes OR diabetes mellitus OR DM OR diabetic) | 229 |
| CNKI/Wanfang | 糖尿病 AND 预算影响分析 | 35 |
